# GENDER DIFFERENCES IN THE ASSOCIATION BETWEEN SLEEP AND INFLAMMATORY MARKERS: RESULTS FROM THE EINSTEIN AGING STUDY

**DOI:** 10.1093/geroni/igad104.1830

**Published:** 2023-12-21

**Authors:** Linying Ji, Yuqi Shen, Jennifer Graham-Engeland, Carol Derby, Christopher Engeland, Orfeu Buxton

**Affiliations:** Montana State University, Bozeman, Montana, United States; Pennsylvania State University, State College, Pennsylvania, United States; Pennsylvania State University, State College, Pennsylvania, United States; Albert Einstein College of Medicine, Bronx, New York, United States; Pennsylvania State University, University Park, Pennsylvania, United States; Pennsylvania State University, State College, Pennsylvania, United States

## Abstract

Prior research links poor sleep and higher systemic inflammation. Gender differences in both sleep and in inflammatory responses have been observed. We investigated gender differences in the association between levels as well as variability of objective sleep health and inflammatory markers (including stimulated inflammatory markers) among a diverse sample of older adults. Participants free of dementia (N=215, Mage=77.5 years, 64% women; 46% White, 39% Black, 15% Hispanic/other) were from The Einstein Aging Study. Actigraphy (2 weeks) yielded wake after sleep onset (WASO), total night sleep time (TST), and midpoint night sleep. Inflammatory markers included circulating C-reactive protein (CRP) and circulating and lipopolysaccharide (LPS)-stimulated levels of interleukin (IL)-1β, IL-4, IL-6, IL-8, IL-10, and tumor necrosis factor (TNF)-𝛼. Bayesian variability models evaluated associations of inflammatory markers with mean and variability of sleep measures, adjusted for BMI, gender, age, education, race/ethnicity. Circulating inflammatory markers were not associated with sleep measures, except higher TST variability with higher circulating IL-6 among women. For stimulated cytokines, among men, higher mean WASO, TST, and later midpoint were associated with higher stimulated IL-8 and TNF-𝛼. All stimulated cytokines except IL-1β were associated with greater variability in WASO, TST, and sleep timing among men. Among women, the only significant association with stimulated cytokines was between higher mean TST and higher stimulated IL-8. We found gender differences in the associations between sleep and inflammation in older adults, primarily in men and for stimulated (not circulating) cytokines. Interestingly, associations were also more frequently observed for sleep variability than mean levels.

